# Community-based COVID-19 active case finding and rapid response in the Democratic Republic of the Congo: Improving case detection and response

**DOI:** 10.1371/journal.pone.0278251

**Published:** 2023-05-18

**Authors:** John Otokoye Otshudiema, Gervais Léon Tengomo Folefack, Justus M. Nsio, Cathy H. Kakema, Luigino Minikulu, Aimé Bafuana, Joel B. Kosianza, Antoine K. Mfumu, Edith Nkwembe, Yannick Munyeku-Bazitama, Sheila Makiala-Mandanda, Noé Guinko, Gisèle Mbuyi, Jean-Marie K. Tshilumbu, Guy N. Saidi, Moreau-Serge Umba-di-Masiala, Amos K. Ebondo, Jean-Jacques Mutonj, Serge Kalombo, Jad Kabeya, Taty K. Mawanda, Faustin N. Bile, Gaby K. Kasereka, Placide Mbala-Kingebeni, Steve Ahuka-Mundeke, Humphrey Cyprian Karamagi, Karl Njuwa Fai, Amédée Prosper Djiguimde

**Affiliations:** 1 COVID-19 Response, World Health Organization, Kinshasa, Democratic Republic of the Congo; 2 COVID-19 Response, Ministry of Health, Kinshasa, Democratic Republic of the Congo; 3 COVID-19 Laboratory and Epidemiology Team, National Institute of Biomedical Research, Kinshasa, Democratic Republic of the Congo; 4 Data Analytics and Knowledge Management, World Health Organization Regional Office for Africa, Brazzaville, Democratic Republic of Congo; 5 Research Arm – MSF Epicentre, Yaoundé, Cameroon; Washington State University, UNITED STATES

## Abstract

A community-based coronavirus disease (COVID-19) active case-finding strategy using an antigen-detecting rapid diagnostic test (Ag-RDT) was implemented in the Democratic Republic of Congo (DRC) to enhance COVID-19 case detection. With this pilot community-based active case finding and response program that was designed as a clinical, prospective testing performance, and implementation study, we aimed to identify insights to improve community diagnosis and rapid response to COVID-19. This pilot study was modeled on the DRC’s National COVID-19 Response Plan and the COVID-19 Ag-RDT screening algorithm defined by the World Health Organization (WHO), with case findings implemented in 259 health areas, 39 health zones, and 9 provinces. In each health area, a 7-member interdisciplinary field team tested the close contacts (ring strategy) and applied preventive and control measures to each confirmed case. The COVID-19 testing capacity increased from 0.3 tests per 10,000 inhabitants per week in the first wave to 0.4, 1.6, and 2.2 in the second, third, and fourth waves, respectively. From January to November 2021, this capacity increase contributed to an average of 10.5% of COVID-19 tests in the DRC, with 7,110 positive Ag-RDT results for 40,226 suspected cases and close contacts who were tested (53.6% female, median age: 37 years [interquartile range: 26.0–50.0)]. Overall, 79.7% (n = 32,071) of the participants were symptomatic and 7.6% (n = 3,073) had comorbidities. The Ag-RDT sensitivity and specificity were 55.5% and 99.0%, respectively, based on reverse transcription polymerase chain reaction analysis, and there was substantial agreement between the tests (k = 0.63). Despite its limited sensitivity, the Ag-RDT has improved COVID-19 testing capacity, enabling earlier detection, isolation, and treatment of COVID-19 cases. Our findings support the community testing of suspected cases and asymptomatic close contacts of confirmed cases to reduce disease spread and virus transmission.

## Introduction

Since the first coronavirus disease (COVID-19) case was found in Wuhan, China, on December 21, 2019, the severe acute respiratory syndrome coronavirus 2 (SARS-CoV-2) has spread worldwide [1, 2]. The World Health Organization (WHO) claimed that just 14.2%, or one in seven, of SARS-CoV-2 infections have been detected in Africa [3]. From the pandemic’s beginning until October 10, 2021, African countries have performed more than 70 million COVID-19 tests, representing a small percentage of the continent’s 1.3 billion people. In contrast, the United States, with approximately one-third of Africa’s population, has recorded over 550 million tests, whereas the United Kingdom, with less than 10% of the African population, has conducted over 280 million tests [3].

COVID-19 cases are underreported in most African countries, as detection in most countries has focused on people meeting the clinical case definition and reporting to health facilities, and testing of travelers while ignoring asymptomatic cases that are known to play a significant role in driving transmission [4]. Reverse transcription polymerase chain reaction (RT-PCR) is the reference method for SARS-CoV-2 detection [5, 6]; however, it has restricted availability in most low- and middle-income nations owing to the cost associated with the need for robust laboratory infrastructure and highly trained staff [7]. Challenges associated with RT-PCR availability have led to low testing capacity and difficulties in outbreak management.

On Oct 14, 2021, the WHO Regional Office for Africa recommended the use of an antigen-detecting rapid diagnostic test (Ag-RDT) to improve community screening for COVID-19, which could reach more than seven million people in eight countries [8]. Some studies have indicated a lack of understanding of different types of Ag-RDTs, their adequate usage, and their real-world sensitivity of up to 70% and have elicited concerns associated with Ag-RDT use in large-scale campaigns [9]. However, a recent study on Ag-RDT performance and operational feasibility in a sub-Saharan African country demonstrated the cost-effectiveness of these diagnostic tools for COVID-19 diagnosis, especially when integrated through an effective algorithm [10]. Although asymptomatic infected individuals have a substantially reduced risk of spreading SARS-CoV-2 compared with COVID-19 cases [11–13], a significant proportion of asymptomatic carriers can be identified by actively tracking cases and expanding the testing to close contacts. Appropriate early-stage isolation of infected individuals reduces the risk and magnitude of transmission [14].

Experts agree that the active case finding (ACF) approach, which entails systematic screening and clinical evaluation of individuals with a presumptive diagnosis in a target group using RDTs or other procedures, is crucial to case finding, as shown in several fields [15, 16].

In the Democratic Republic of the Congo (DRC), COVID-19 diagnosis using RT-PCR was only conducted at the National Institute of Biomedical Research (INRB) and a few laboratories that were concentrated in cities, leading to a prolonged turnaround time to obtain results as well as low testing capacity, estimated at 0.3 tests, in contrast to a minimum of 10 tests, per 10,000 persons per week during the first wave [17].

The DRC was one of the first African countries to implement a community-based ACF approach using Ag-RDT, beginning in January 2021. In the national laboratory diagnosis plan developed in response to COVID-19 during the early stages of the epidemic in the DRC, Ag-RDTs were recommended as alternative screening methods. In response to the threat that COVID-19 poses to public health and with the collaborative effort of the WHO, the Gavi, the vaccine alliance; and the FIND, the global alliance for diagnostics, the DRC Ministry of Health implemented a comprehensive community-based ACF testing strategy using Ag-RDTs among suspected COVID-19 cases and close contacts of confirmed cases in 39 active health zones (HZ).

In many African countries, there is a paucity of information on community-based testing strategies, SARS-CoV-2 symptoms, and the effectiveness of rapid antigen tests in the field. In this study, we share the DRC’s initial experience in scaling up community-based COVID-19 ACF using Ag-RDTs and organizing an integrated response around each new case or cluster to prevent further virus transmission. We aimed to identify the best practices and lessons learned to improve SARS-CoV-2 detection and Ag-RDT-based implementation of a rapid response to COVID-19. Furthermore, we compared the testing capabilities and field performance of Ag-RDTs with those of SARS-CoV-2 RT-PCR, the gold standard.

## Materials and methods

### Study design and population

This prospective clinical study investigated the early detection and implementation of containment protocols for COVID-19 cases. All the study participants, regardless of age, met the DRC requirements for the COVID-19 case definition.

### Case definition

We used the COVID-19 case definition of the DRC Ministry of Health to classify cases. A confirmed case was defined as any person with an RT-PCR- or Ag-RDT-confirmed SARS-CoV-2 infection, irrespective of clinical signs and symptoms. A suspected COVID-19 case was defined in two ways: 1) an individual with one or more signs of an acute respiratory infection, regardless of illness severity, and 2) anyone who had close contact with a confirmed case, regardless of whether they had COVID-19 symptoms themselves.

### Study settings and interdisciplinary teams

This pilot program was implemented in 39 active HZ within the 9 most afflicted of the 26 provinces.

In the initial phase, we implemented an Ag-RDT-based ACF approach in 16 HZ. An additional 23 HZ were included in the second (extension) phase. Within the HZ, laboratory technicians and end users received training on Ag-RDT screening during training sessions conducted across 259 health areas per standard operating procedures, training modules, and technical guides. The training materials included presentations, demonstrations, and good laboratory practice. Both provincial laboratories and end users undertook quality control at the operational level. All HZ submitted weekly monitoring reports on Ag-RDT inventories, personal protective equipment, and consumables.

Real-time sharing of daily case notification reports and weekly situation reports facilitated daily monitoring and oversight of ongoing ACF efforts. At each targeted health area or community site, we trained and established a 7-member field interdisciplinary team (7-FIT) to conduct ACF that included Ag-RDT testing with isolation, timely case management, and contact tracing and testing of suspected COVID-19 cases. The 7-FIT team was composed of a field epidemiologist or surveillance officer to ensure proper investigation and contact listing, a laboratory technician for respiratory sample collection and testing, a communication officer to ensure community adherence to services, a psychologist to prepare suspected cases for laboratory result announcement, an infection prevention and control agent to ensure proper patient isolation and decontamination of all places visited by each confirmed case during the last 2 days, a clinician to ensure proper evaluation and timely case management, and a community site coordinator.

### Data collection

From January through November 2021, we enrolled and tested COVID-19 suspected cases at targeted health facilities or communities that were distributed in 39 active and hotspot HZs in 9 affected provinces. COVID-19 suspected cases included individuals who reported symptoms consistent with the COVID-19 case definition, close contacts of confirmed cases, healthcare workers, and first responders, regardless of their clinical status. Data on individual age and sex, comorbidities, presence or absence of symptoms, and date of occurrence of symptoms or signs were collected using a standardized questionnaire. Moreover, through routine surveillance, we collected data on the total number of screened individuals and COVID-19 cases identified at the national level.

COVID-19 suspected cases were isolated for 14 days under household quarantine according to home-based COVID-19 management guidelines and received over-the-counter medication or medical referrals in case of severe COVID-19.

### Description of COVID-19 ACF strategy in the community

Using the ring strategy, a 7-FIT that targeted all individuals living or working in close contact (within a 100 m radius) surroundings of each newly confirmed case, comprehensively implemented the following interventions: Ag-RDT testing on a voluntary basis, facilitating risk communication, infection prevention and control measures for isolation and home-based treatment, transfer of contacts or suspected cases with a positive Ag-RDT result to a COVID-19 treatment center, isolation, and home-based treatment, or transfer to a COVID-19 treatment center to prevent further infection spread by interrupting the chain of transmission. The contacts of each newly confirmed case were listed and identified by defining the established community and therapeutic itinerary of each newly confirmed case during the last 2 days prior to notification or confirmation. This allowed the 7-FIT to conduct ACF, testing, treatment, and control measures for each case’s household, including neighbors, classmates, co-workers, friends, health providers, caregivers, or any individual who interacted directly or indirectly with the case within two days before symptom onset or sample collection and confirmation (if asymptomatic). In addition, contacts included close participants of a mass-gathering event or co-passengers in an aircraft or vehicle, as well as co-detainees of a prison. A hygiene pack, including face masks and hand sanitizers, was distributed to each household, targeted by the ACF and the response strategy. Those who tested negative received a flyer with prevention measures and a phone number to call in case of symptom occurrence. No daily follow-ups were required. Positive contacts were referred to COVID-19 treatment centers if symptomatic, or they received home-based care if asymptomatic.

### Description of the COVID-19 ACF strategy in the health facility

The 7-FIT team visited health facilities once a week to review the registers of admission and discharge to identify those who met the COVID-19 case definition and were not reported as suspected cases. These suspected cases and their direct and indirect contacts were followed up in the community for further screening and testing 14 days after their contact with a primary case. Confirmed COVID-19 cases were referred to treatment centers or benefitted from home-based care, depending on their clinical status.

### Testing strategy

**Fig 1:**
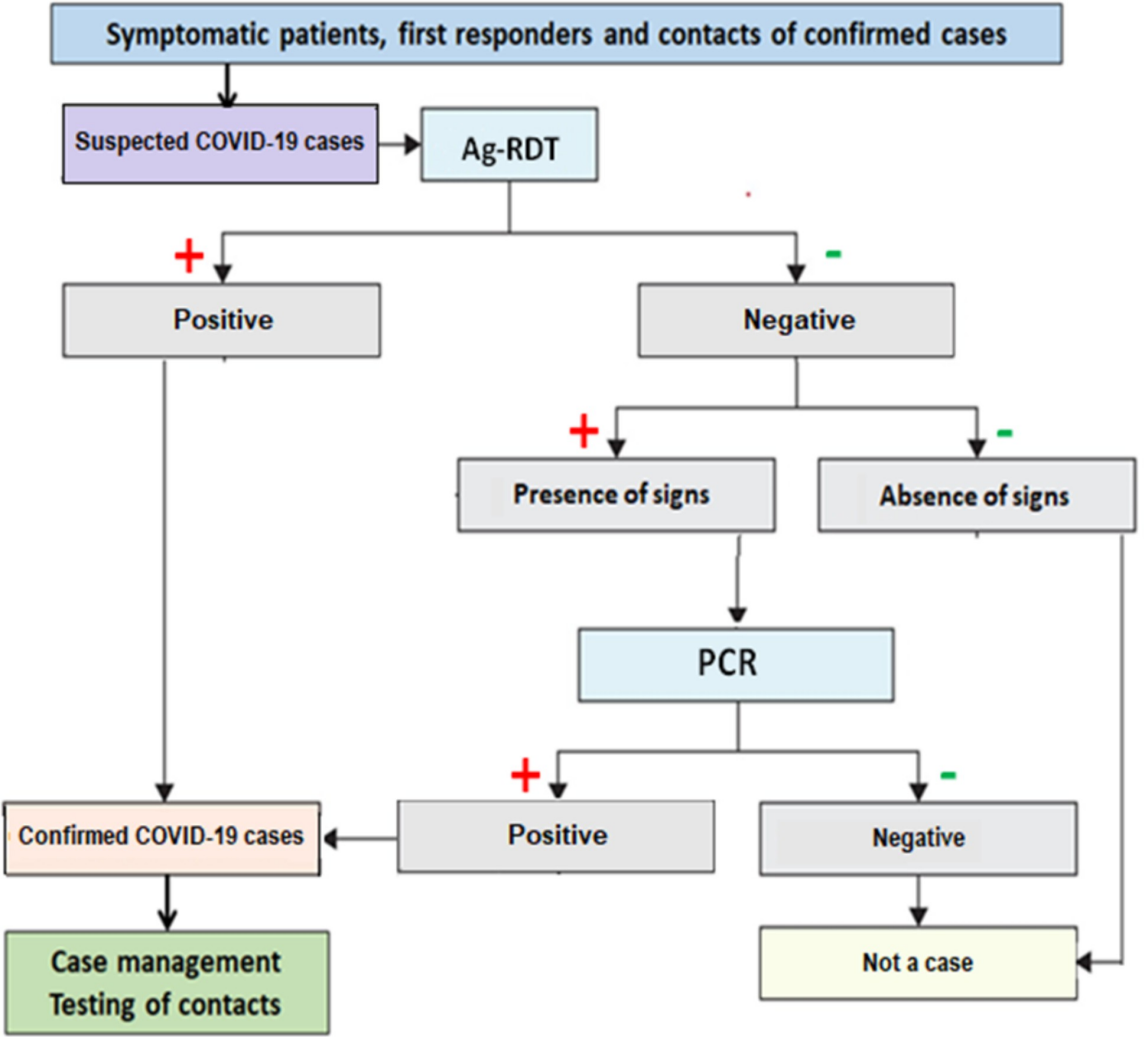
Algorithm for SARS-CoV-2 diagnosis using Ag-RDT and RT-PCR. SARS-CoV-2, severe acute respiratory syndrome coronavirus 2; Ag-RDT, antigen-detecting rapid diagnostic test; RT-PCR, reverse transcription polymerase chain reaction.

We used an optimized diagnosis algorithm (Fig 1) that included re-sampling symptomatic cases with negative Ag-RDT results for confirmation by RT-PCR.

The procedures for sample collection, packaging, and delivery to a laboratory setting followed national and international guidelines and the test kit manufacturers’ instructions [18–20]. A set of three nasopharyngeal or oropharyngeal swabs were collected from suspected cases and their close contacts by trained laboratory technicians. One set of swabs was tested for SARS-CoV-2 using the Panbio^™^ COVID-19 Ag Rapid Test Device (Abbott Rapid Diagnostics, Jena, Germany; sensitivity: 91.4%; specificity: 99.8%) or STANDARD^™^ Q COVID-19 Ag Test (SD Biosensor, Inc., Suwon-si, Gyeonggi-do, Korea; sensitivity: 76.6%; specificity: 99.3%) at the point of sample collection, according to the manufacturer’s instructions [19, 20]. The remaining set of collected swabs was triple-packed and transported in a sterile container using the BD universal viral transport system (Becton, Dickinson, and Company), ensuring conditions for secure transportation and the maintenance of adequate and stable temperatures (2–4°C). The samples were then stored at –20°C for subsequent molecular analyses.

A SARS-CoV-2 RT-PCR confirmatory test was conducted on Ag-RDT-negative symptomatic participants and on a subset of Ag-RDT-positive participants for the test kit performance assessment. All RT-PCR assays were conducted in accordance with the WHO interim guidance and the manufacturer’s instructions. We used the Daan Gene Nucleic Acid Extraction Kit (Daan Gene Co., Ltd., Sun Yat-sen University, Guangzhou) to obtain viral RNA as per the manufacturer’s instructions [21]. Then RT-PCR was performed on RNA extracts using a manual kit [Daan Gene Detection Kit for 2019 Novel Coronavirus (2019-nCoV) RNA (PCR-Fluorescence Probing), Daan Gene Co., Ltd., Sun Yat-sen University, Guangzhou]. The Daan Gene RT-PCR kit detects the SARS-CoV-2 nucleocapsid protein (N) and ORF1ab gene sequences through FAM and VIC/HEX labeled probes, respectively. Additionally, the kit uses the human housekeeping gene RNP (Ribonuclease P) as a target gene for the internal control, whose probe is Cy5-labeled, as well as positive and negative controls. The ABI 7500 (Applied Biosystems, Beverly, MA, USA) was used as the amplification and detection instrument. A sample was considered positive for SARS-CoV-2 if the test result showed an amplification curve in the FAM and/or VIC channels with Ct values ≤ 40 and no amplification or a Ct value ≤ 40 for the Cy5 channel. Samples with no amplification curve in the FAM and VIC channels and a Ct value ≤ 40 for the Cy5 channel were considered negative. The test was repeated for all samples meeting neither the positivity, nor the negativity criteria. For quality control, both the negative and positive controls were used for each run. The run was valid when there was no obvious amplification curve for FAM and VIC detection channels and the Cy5 channel’s Ct value was <35 for the negative control. In addition, for the positive control, the run was valid when there were obvious amplification curves for the FAM and VIC detection channels and a Ct value ≤ 32, and an amplification curve or no amplification curve for the Cy5 channel.

This operational research study was aligned with routine COVID-19 surveillance. In conformance with prevailing ethical considerations for epidemiological research during the pandemic, individual consent was not required or applicable. The Kinshasa School of Public Health Ethics Committee approved this study as a routine public health emergency response practice (ESP/CE/64/2021; April 13, 2021).

### Statistical analysis

Percentages were used to describe categorical variables, whereas medians and interquartile ranges (IQR) were used to summarize continuous variables when applicable. Depending on the data distribution, we employed the Student’s *t*-test or Mann–Whitney *U* test for univariate comparisons. Chi-square and Fisher’s exact tests were used to analyze categorical variables. The proportions of performed SARS-CoV-2 diagnostic tests and COVID-19 cases detected using the Ag-RDT were estimated based on national data within the same period. The SARS-CoV-2 positivity rate was calculated as the number of identified cases (Ag-RDT positive) divided by the total number of suspected cases and close contacts. Furthermore, the positivity rate was estimated separately according to age group and was interpreted as the secondary attack rate. Ag-RDT (Both the Panbio^™^ COVID-19 and STANDARD^™^ Q COVID-19) diagnostic test sensitivities and 95% confidence intervals were calculated as the proportion of positive results among RT-PCR-confirmed SARS-CoV-2 infections. Specificity was estimated as the proportion of negative results among patients who tested negative by RT-PCR. Cohen’s kappa coefficient (κ) was used to quantify the agreement between Ag-RDT (both the Panbio^™^ COVID-19 and STANDARD^™^ Q COVID-19) diagnostic tests and the gold standard RT-PCR. Performance characteristics were estimated based on the presence or absence of symptoms. All analyses were performed using R Statistical Software (version 4.0.3; R Core Team 2021).

## Results

### Active detection of cases by scaling up the use of Ag-RDTs in the community

**Fig 2:**
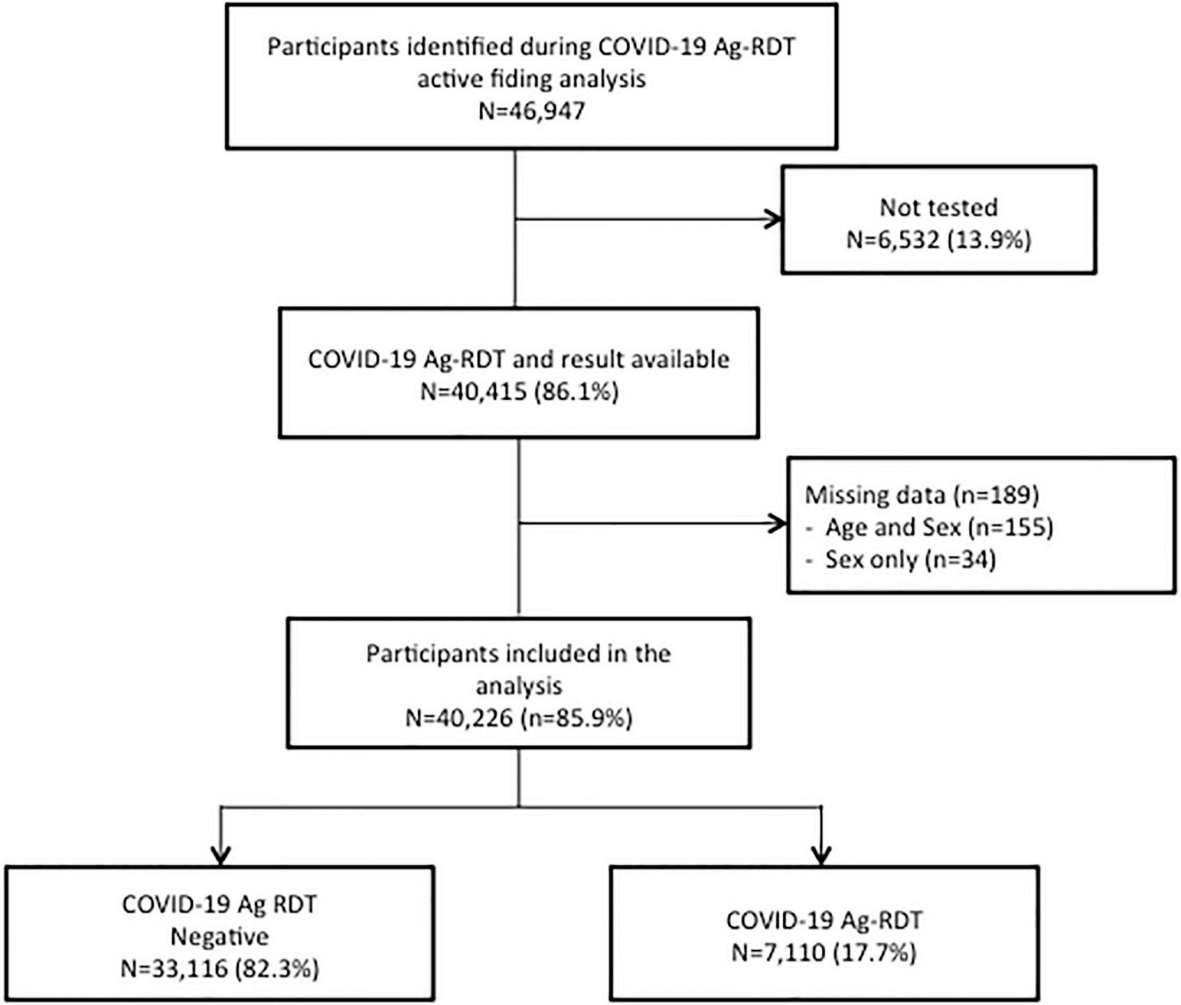
Flow chart showing the selection of participants for the analysis.

During the study period, 46,947 suspected cases and their close contacts were identified in the database; however, only 40,226 cases were included in our analysis because of the availability of test results, age, and sex information (Fig 2).

### Characteristics of the study population

Of the 40,226 participants, 53.6% were female. The median age was 37 years (IQR: 26.0–50.0), which was significantly higher among participants with positive COVID-19 Ag-RDT results (Table 1). Approximately 6.6% and 8.8% of the participants were aged <15 and ≥65 years, respectively. Healthcare workers represented less than 5% of identified participants, and their proportion was significantly lower among COVID-19 positive cases compared with the negative cases (1.7% vs. 3.4%; *p*<0.001). Nearly half of the participants that were identified and included were from Kinshasa province, which comprises one-third of the HZ (Table 1).

**Table 1:** Sociodemographic characteristics and type of suspected cases according to the SARS-CoV-2 test result in the DRC from January to November 2021.

Characteristics	SARS-CoV-2 Ag-RDT result		Total	*p*-value
	Negative N = 33,116 n (%)	Positive N = 7,110 n (%)	N = 40,226 n (%)	
**Sex**				**0.128**
Male	17,708 (53.5)	3,873 (54.5)	21,581 (53.6)	
Female	15,408 (46.5)	3,237 (45.5)	18,645 (46.4)	
**Age** (years, N = 39,017)				**<0.001**
Age, mean (SD)	37.9 (17.4)	41.7 (17.7)	38.6 (17.5)	
Age, median (IQR)	36.0 (25.0–49.0)	40.0 (28.0–54.0)	37.0 (26.0–50.0)	**<0.001**
**Age group, years**				
<15	2,315 (7.2)	258 (3.8)	2,573 (6.6)	
15–25	5,021 (15.6)	904 (13.1)	5,925 (15.8)	
25–35	7,418 (23.1)	1,474 (21.4)	8,892 (28.2)	
35–50	9,369 (29.2)	2,055 (29.9)	11,424 (29.3)	
50–65	5,409 (16.8)	1,354 (19.7)	6,763 (17.3)	
≥65	2,605 (8.1)	835 (12.1)	3,440 (8.8)	
*Province (N = 40,182)				**<0.001**
KINSHASA	16,930 (51.2)	2,761 (38.9)	19,691 (49.0)	
KONGO CENTRAL	5,264 (15.9)	658 (9.3)	5,922 (14.7)	
NORD KIVU	4,353 (13.2)	1,097 (15.4)	5,450 (13.6)	
SUD KIVU	4,884 (14.7)	1,506 (21.2)	6,390 (15.9)	
Other	1,647 (5.0)	1,082 (15.2)	2,729 (6.8)	
**Healthcare workers** (N = 39,361)				**<0.001**
No	31,151 (96.3)	6,886 (98.3)	38,037 (96.6)	
Yes	1,204 (3.7)	120 (1.7)	1,324 (3.4)	
**Category of participants** (N = 40,068)				**<0.001**
Travelers	166 (0.5)	3 (0.0)	169 (0.4)	
Asymptomatic contact	5,612 (17.0)	591 (8.3)	6,203 (15.5)	
Symptomatic contact	27,198 (82.5)	6,498 (91.6)	33,696 (84.1)	

* Other: HAUT KATANGA, GOMA, and TSHOPO

SARS-CoV-2, severe acute respiratory syndrome coronavirus 2; DRC, Democratic Republic of Congo; Ag-RDT, antigen-detecting rapid diagnostic test; SD, standard deviation; IQR, interquartile range

Overall, 79.7% (n = 32,071) of the participants manifested at least one symptom, with a higher proportion of symptomatic people among the Ag-RDT-positive participants (90.2% vs. 78.7%, p<0.001) (Table 2). The median time from symptom onset to sample collection for diagnosis was 4 days (IQR: 2–7). Among symptomatic participants with positive Ag-RDT results, the most frequent signs and symptoms were cough (65.2%), dyspnea (65.2%), headache (43.5%), fever (43.1%), asthenia (43.1%), rhinorrhea (41.5%), anosmia (24.9%), and sore throat (14.3%) (Table 2). Similar proportions of asthenia and sore throat were observed among participants with positive and negative Ag-RDT results. Approximately 7.6% of participants had at least one comorbidity, with a significantly higher proportion of participants with positive Ag-RDT results. Less than 1% of patients had two or more comorbidities (Table 2).

**Table 2:** Clinical signs, symptoms, and pre-existing conditions by the SARS-CoV-2 test result in the DRC from January to November 2021.

Characteristics	Covid-19 Ag-RDT result		Total	p-value
	Negative	Positive		
	N = 33 116 n (%)	N = 7 110 n (%)	40 226	
**Clinical signs and symptoms**				
Sore throat (N = 39 099)	**32 081**	**7 018**		0.872
Yes	4 572 (14.3)	1 006 (14.3)	5 578 (14.3)	
Rhinorrhea (N = 39 260)	**32 222**	**7 038**		<0.001
Yes	7 971 (24.7)	2 924 (41.5)	10 895 (27.8)	
Cough (N = 39 432)	**32 373**	**7 059**		<0.001
Yes	13 810 (42.7)	4 604 (65.2)	18 414 (46.7)	
Dyspnea (N = 39 432)	**32 204**	**7 020**		0.159
Yes	13 810 (42.7)	4 604 (65.2)	18 414 (46.7)	
Fever (N = 39 484)	**32 441**	**7 043**		<0.001
Yes	12 545 (38.7)	3 036 (43.1)	15 581 (39.5)	
Asthenia (N = 39 484)	**32 148**	**7 021**		0.971
Yes	12 545 (38.7)	3 036 (43.1)	15 581 (39.5)	
Headaches (N = 39329)	**32 314**	**7 015**		<0.001
Yes	9 354 (28.9)	3 052 (43.5)	12 406 (31.5)	
Myalgia (N = 39 197)	**32 187**	**7 010**		<0.001
Yes	3 764 (11.7)	1 045 (14.9)	4 809 (12.3)	
Anosmia (N = 39 162)	**32 157**	**7 005**		<0.001
Yes	3 556 (11.1)	1 745 (24.9)	5 301 (13.5)	
Symptom (N = 39 706)	**32 626**	**7 080**		<0.001
Yes	25 688 (78.7)	6 383 (90.2)	32 071 (80.8)	
**Pre-existing conditions**				
Diabetes (N = 36 072)	**29 359**	**6 713**		<0.001
Yes	1 398 (4.8)	1 178 (17.5)	2 576 (7.1)	
Hypertension (N = 36 085)	**29 374**	**6 711**		0.001
Yes	373 (1.3)	122 (1.8)	495 (1.4)	
Cardiovascular (N = 36 068)	**29 358**	**6 710**		0.541
Yes	165 (0.6)	33 (0.5)	198 (0.5)	
Asthma (N = 36 071)	**29 361**	**6 710**		0.486
Yes	144 (0.5)	38 (0.6)	182 (0.5)	
Cancer (N = 36 068)	**29 358**	**6 710**		<0.001
Yes	10 (0.0)	10 (0.1)	20 (0.06)	
Renal issues (N = 36 069)	**29 358**	**6 711**		0.271
Yes	18 (0.1)	7 (0.1)	25 (0.07)	
Comorbidities (N = 40 226)	**37 152**	**3 073**		<0.001
Yes	1 771 (5.3)	1 302 (18.3)	3 073 (7.6)	
Number of comorbidities (N = 40226)	**33 116**	**7 110**		<0.001
0	31 345 (94.6)	5 808 (81.7)	37 153 (92.4)	
1	1 515 (4.6)	1 240 (17.4)	2 755 (6.8)	
2 or more	256 (0.8)	62 (0.9)	318 (0.8)	

SARS-CoV-2, severe acute respiratory syndrome coronavirus 2; DRC, Democratic Republic of Congo; Ag-RDT, antigen-detecting rapid diagnostic test

### Contribution of the Ag-RDT case-finding strategy to the diagnosis of COVID-19

**Fig 3:**
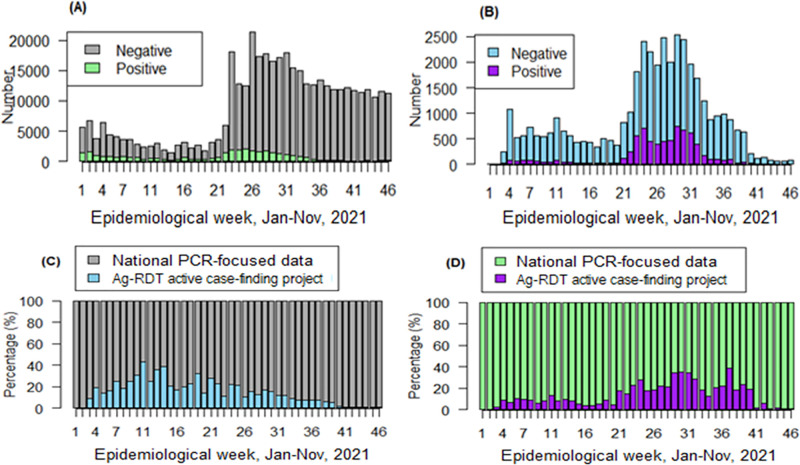
Contribution of the Ag-RDT case-finding strategy to the diagnosis of COVID-19. (A) Total SARS-CoV-2 diagnostic tests carried out and test results by epidemiological week at the country level. (B) Ag-RDT test results by epidemiological week at the study site. (C) The proportion of Ag-RDT tests and (D) proportion of positive Ag-RDT tests among total cases identified at the country level. SARS-CoV-2, severe acute respiratory syndrome coronavirus 2; Ag-RDT, antigen-detecting rapid diagnostic test; COVID-19, coronavirus disease.

Nasopharyngeal swabs were collected from 40,226 suspected cases and close contacts of the confirmed cases. Overall, 7,110 cases were identified as being SARS-CoV-2 infected, leading to an Ag-RDT positivity rate of 17.7% (95% CI: 17.3–18.1), which significantly increased with age (10.0% for ages <15 years and 24.3% for age ≥65 years). Almost all results (99%) were delivered within 24 h after sample collection. At the national level, the case-finding strategy using Ag-RDT contributed to an average of 10.5% of SARS-CoV-2 tests carried out in the DRC and 17.7% of new cases in the community during the study period. These figures substantially increased during the thi^rd^ wave of the pandemic (weeks 21 to 37) within the study period, with 12.7% (28,235/222,920) of Ag-RDTs performed and 24.0% (6,293/26,234) of new COVID-19 cases detected on average (Fig 3). The remaining COVID-19 cases were detected during the surge through routine surveillance and among travelers at 60.6% (n = 15,908) and 15.4% (n = 4,033), respectively.

### Overall testing capacity

Table 3 shows how the overall SARS-CoV-2 testing capacity increased over time, from 0.3 to 0.4 tests per 10,000 inhabitants per week in the first and second waves to 1.6 and 2.2 tests per 10,000 inhabitants in the third and fourth waves, respectively, after the rollout of Ag-RDTs. During the third and fourth waves, the proportion of Ag-RDTs among confirmatory tests was 15.5%.

**Table 3:** Evaluation of SARS-CoV-2 testing capacity during the four waves of the COVID-19 resurgence in the DRC.

	1^st^ wave	2^nd^ wave	3^rd^ wave	4^th^ wave	*P-Value*					
					(1 vs. 2)	(1 vs. 3)	(1 vs. 4)	(2 vs. 3)	(2 vs. 4)	(3 vs. 4)
Wave, EW*	EW16/2020–EW34/2020	EW47/2020–EW13/2021	EW20/2021–EW32/2021	EW45/2021–EW52/2021						
**Wave, period**	Apr 13–Aug 23, 2020	Nov 16, 2020–Apr 04, 2021	May 17, 2021–Aug 15, 2021	Nov 08, 2021–Jan 02, 2022						
**SARS-CoV-2 tests performed (PCR and Ag-RDT)**	56,637	66,560	157, 945	163,942						
**SARS-CoV-2 tests/10,000 inhabitants/week, Median (IQR)**	0.3 (0.1–0.6)	0.4 (0.2–0.6)	1.6 (0.3–2.1)	2.2 (1.4–3.3)	0.0032	<0.001	<0.001	<0.001	<0.001	<0.001

A P-value <0.001 means that the difference was statistically relevant. MedCalc uses the ‘‘N-1” Chi-square test as recommended by Campbell (2007) and Richardson (2011). SARS-CoV-2, severe acute respiratory syndrome coronavirus 2; COVID-19, coronavirus disease; DRC, Democratic Republic of Congo; Ag-RDT, antigen-detecting rapid diagnostic test; PCR, transcription polymerase chain reaction; IQR, interquartile range.

### Enabling access to reliable SARS-CoV-2 testing to people in hard-to-reach communities

The program reached approximately 5 million people distributed in 39 HZ in 9 provinces of the DRC. During the two phases of the program, 39 HZs held 76 training sessions, with a maximum of 20 participants each. Thus, 1,515 laboratory technicians and end users were trained (healthcare providers, field epidemiologists, and community health workers). During phase one, 30 national laboratory technicians and 26 national supervisors were trained, and five national supervisors completed the online international WHO course on Ag-RDTs. Moreover, 80,000 Ag-RDTs (Panbio^™^ COVID-19 Ag-RDT and STANDARD^™^ Q COVID-19 Ag-RDT) were distributed throughout the 39 HZ after quality control was performed by INRB Kinshasa.

Furthermore, at least 25,000 copies of case-finding Ag-RDT guides, standard operating procedures, and algorithms, along with 200,000 posters of the COVID-19 case definition, were distributed, with 80 tablets configured with the Early Warning, Alert, and Response System (EWARS) application, and supplies of communication credits and Internet bundles. In all nine provinces that were the focus of the initiative, 14 missions were set up by national specialists from the Ministry of Health, the National Reference Laboratory (INRB), and WHO to ensure quality assurance and formative supervision.

### Performance of SARS-CoV-2 Ag-RDT compared with RT-PCR

A total of 3,225 nasopharyngeal swabs from suspected cases and close contacts were tested using approved confirmatory tests (Ag-RDTs) and RT-PCR (gold standard), of which 687 and 2,538 samples were RT-PCR positive and negative, respectively. Of the 414 positive and 2,811 negative results generated by Ag-RDTs, 35 (8.5%) were false positives, and 308 (11.0%) were false negatives (sensitivity 55.5% [95% CI: 51.4–58.9], specificity 99.0% [95% CI: 98.1–99.0], positive predictive value 91.5% [95% CI: 88.4–94.0], and negative predictive value 89.0% [95% CI: 87.8–90.2]). The level of agreement between the two tests was 0.63. Sensitivity was the highest among symptomatic COVID-19 cases compared to asymptomatic carriers (59.7% vs. 44.8%; *p*<0.001).

### Improved Ag-RDT performance with RT-PCR test for negative Ag-RDT samples of symptomatic participants

A total of 2,221 symptomatic participants with negative Ag-RDTs results were subsequently tested using RT-PCR, of whom 211 (9.5%) were RT-PCR positive, were classified as confirmed cases, and received treatment. Thus, this diagnosis strategy improved the testing capacity to identify COVID-19 cases, with a sensitivity of 85.9% (95% CI: 83.0–88.4) and a positive predictive value of 94.4% (95% CI: 92.3–96.1) (Table 4).

**Table 4:** Ag-RDT performance characteristics using RT-PCR as the gold standard in Ag-RDT case finding strategy in the DRC from January to November 2021.

	Sensitivity (95% CI)	Specificity (95% CI)	Predictive values		True	False	True	False	Coefficient
			PPV (95% CI)	NPV (95% CI)	Positive	Positive	Negative	Negative	Kappa
Total (n = 3225)	0.55 (0.51–0.59)	0.99(0.98–0.99)	0.92(0.89–0.94)	0.89(0.88–0.90)	379	35	2,503	308	0.63
Symptomatic (n = 2553)	0.59(0.55–0.63)	0.99 (0.98–0.99)	0.92 (0.89–0.95)	0.90 (0.89–0.92)	304	28	2,010	211	0.66
Asymptomatic (n = 635)	0.45 (0.37–0.53)	0.99(0.97–1.0)	0.91 (0.85–0.97)	0.84(0.81–0.87)	73	7	466	89	0.52

PPV: positive predictive value; NPV: negative predictive value; CI, Confidence interval; DRC, Democratic Republic of Congo; Ag-RDT, antigen-detecting rapid diagnostic test; RT-PCR, reverse transcription polymerase chain reaction

## Discussion

### Study implementation and key findings

This innovative pilot program for COVID-19 ACF using SARS-CoV-2 Ag-RDTs for screening, both in health facilities and throughout the communities of 39 HZs located in 9 provinces in the DRC, was successfully implemented. This program achieved the decentralization of SARS-CoV-2 detection to hard-to-reach communities and included the provision of training to technicians and end users on the correct use of the Ag-RDTs as part of a SARS-CoV-2 testing algorithm, which integrates the Ag-RDTs, establishment of integrated investigation teams, and community sensitization on SARS-CoV-2 infection prevention and management. Overall, the use of Ag-RDTs has effectively contributed to 10.5% of all tests conducted and the early detection of 17.7% of all COVID-19 cases reported in the country from January to November 2021. These findings suggest that suspected cases and asymptomatic close contacts of the patients with COVID-19 included in this study were at a high risk of infection. Furthermore, the implementation of the ACF approach could help scale up SARS-CoV-2 testing and improve case-detection rates in the community. The theory that transmission can be reduced by finding and managing cases, and then tracking down and quarantining their close contacts, is supported by the fact that symptomatic cases are easy to diagnose with tests that are readily available, sensitive, and specific, while asymptomatic cases are unlikely to be identified.

In the absence of sufficient vaccination, proactive community testing is particularly crucial for reducing transmission in African countries with a relatively young population and a high prevalence of silent illnesses. Estimates suggest that 65–85% of SARS-CoV-2 infections in Africa are either asymptomatic or cause minor symptoms [11–13]. Thus, most infected Africans do not seek treatment in local health facilities where most tests are conducted. However, asymptomatic individuals play a key role in facilitating transmission to vulnerable individuals, who can suffer from severe diseases leading to death [3].

In October 2021, the WHO Regional Office for Africa (AFRO) initiated a project in Burundi, Côte d’Ivoire, the Democratic Republic of the Congo, Guinea-Bissau, Mozambique, the Republic of the Congo, Senegal, and Zambia to improve COVID-19 community screening in Africa. The initiative is expected to reach approximately seven million people over 12 months.

The program seeks to enhance the testing capacity by 40% in each participating country. Prior to program commencement, 20 out of 47 countries in the region had not met the WHO-recommended benchmark of 10 tests per 10,000 people each week, and only 4.2% or one in seven COVID-19 cases, were detected [17]. The WHO provided USD 1.8 million to launch this initiative at the regional level. Developed in the late twentieth century for smallpox eradication and used in current Ebola outbreaks in West Africa and the DRC, the "ring strategy" targets those living or working within a 100-m radius of each new confirmed case to prevent infection transmission. This approach helped to successfully test and vaccinate the majority of close contacts of people who were most likely to be infected [17].

According to the WHO, two important community-based interventions that can help reduce the burden of neglected tropical diseases are the implementation of ACF campaigns and mass prophylaxis [22]. Furthermore, the WHO demonstrated that the ACF program is effective in reducing TB in the short term and can substantially reduce patient-incurred costs, which contribute to the end-TB strategy target [23, 24].

The positivity rate observed in our study was comparable to the findings of a similar study conducted in Shanghai, where 20.0% of close contacts were subsequently confirmed to have SARS-CoV-2 infection [25]. Lower positivity rates were reported in early surveillance data from Rwanda and Uganda; 2% and 13% (N = 46,768) of COVID-19 cases were observed, respectively, by November 2020 among contacts who had completed their 14-day follow-up [26]. Concerning household transmission of SARS-CoV-2, reported secondary attack rates are substantially heterogeneous, ranging from 4.6% to 90.0%, with a pooled rate of 27% (95% CI: 21–32%), and adults are more vulnerable than children [27]. The latter phenomenon was observed in our results, where the Ag-RDTs positivity rate was lower among children than adults, suggesting that children might be less susceptible to SARS-CoV-2 infection than adults [16, 28, 29]. As adults are often index cases in household clusters owing to their higher number of social contacts, the positivity rate among children could be considered an indicator of SARS-CoV-2 transmission, especially if there are strict population movement restrictions [28].

The median time from the self-reported onset of symptoms to sample collection for diagnosis was 4 days (IQR: 2–7 days), and almost all results (99%) were delivered within 24 hours. A similar study conducted in Shanghai reported a median result time of 2 days [25]. In studies that were conducted using routine surveillance data, the median (IQR) number of days elapsed from illness onset to sample collection was 7 days (range, 2–17 days), with a median turnaround time of 2 (1–4) days in Nigeria [30], 6 days (IQR: 3–9) in Cameroon [31], and 6 days in Shanghai [25]. Compared with routine surveillance data, the ACF approach significantly reduced the time from symptom onset to diagnosis and shortened the turnaround time, leading to early case management. The study results are noteworthy because they show a shorter interval between the onset of COVID-19 symptoms and treatment initiation. This was advantageous for both populations and individuals because it slowed the spread of the disease and prevented secondary cases, which further helped reduce morbidity and mortality [32–34].

Based on our results, the sensitivity and specificity of the Ag-RDTs were 55.5% and 99.0%, respectively. This low sensitivity indicates that a high proportion of false-negative participants did not receive mitigation measures to prevent SARS-CoV-2 transmission. Field reports suggest that the real-world sensitivity of Ag-RDTs may not exceed 70% [9, 35]. Generally, COVID-19 patients have undetectable antigens one week after symptom onset, whereas RNA remains positive in most cases, which coincides with the infectious period [33]. We did not observe any significant difference in the delay from self-reported symptom onset and sample collection for diagnosis between symptomatic true-positive and symptomatic false-negative participants. The evaluation conducted by FIND concluded that the performance of Ag-RDT depends on the composition of the evaluation panel and viral load in the specimens [35]. The testing algorithm (Fig 1) for resampling cases with symptoms and negative Ag-RDT results to confirm with PCR helped to optimize the performance of the above-mentioned testing strategy, which increased the sensitivity to 85.6%. Combining antigenic tests with molecular tests to improve the detection of active infection is an approach that has been used for other viral infections, such as dengue [36, 37], and yielded new cases but had RT-PCR-related limitations. Overall, the program successfully increased the SARS-CoV-2 detection rate, which will ultimately help the country better control the pandemic, as the program identifies those who have the virus and links them with treatment avenues to prevent further infection transmission. With high specificity, this program also plays an important role in identifying those who do not have COVID-19, thereby allowing them to resume their activities [35].

The proportion of asymptomatic SARS-CoV-2-infected participants included in the study was estimated at approximately 20%, which is consistent with the global proportion of 11.1% during the third wave of the COVID-19 pandemic in the DRC [38]. However, this proportion could be considered low and far from what one would expect following an ACF, which helps identify cases earlier.

### Strengths and limitations

This study had several strengths. This innovative pilot study covered multiple HZ in nine provinces to improve COVID-19 case detection and limit transmission. The contacts of the identified cases were easily identified at the time of diagnosis of the index case, particularly those living in the same household as the cases. However, this study had some limitations. Costs related to ACF interventions raise questions of cost-effectiveness, affordability, and sustainability. The lack of data concerning the index cases prevented estimating the epidemiological parameters of interest. Misclassification of participants due to imperfect sensitivity may lead to underestimating transmission risk. It is difficult to evaluate the individual field performance of Panbio^™^ COVID-19 and STANDARD^™^ Q COVID-19 Ag-RDTs compared to the RT-PCR as both Ag-RDTs were utilized, and the notification system did not include a variable to distinguish which Ag-RDT was used. Some SARS-CoV-2 infected individuals did not provide information about their close relationships, nor were all contacts reachable or ready to cooperate with study requirements, which may lead to representativeness-related issues. In addition, not everyone who tested positive could be isolated on request. As individuals themselves reported the symptoms, there could have been recall bias or reluctance to disclose symptoms during testing. Despite these limitations, our findings from a community-based COVID-19 case study using Ag-RDT provide insights into a simple approach for reducing infection transmission in future COVID-19 surges or pandemics.

## Conclusions

Community-based ACF using the Ag-RDTs pilot program has been successfully implemented in the DRC and has thereby provided access to reliable SARS-CoV-2 testing to people in hard-to-reach communities. This approach effectively contributed to increasing the testing capacity as well as the number of cases detected earlier in the course of the disease, their isolation, and the initiation of appropriate COVID-19 treatment. This pilot program highlights three best practices namely the emphasis on the ACF at the community level with an equipped and trained interdisciplinary team at the HZ level; testing all close contacts regardless of their clinical status; and the field demonstration of the use of an algorithm that combines Ag-RDT and PCR to improve SARS-CoV-2 diagnostic performance. Our findings guide our advocacy for community testing, including asymptomatic close contacts, to curb the spread of the disease and reduce community-based virus transmission.
